# Ellagic acid effects on testis, sex hormones, oxidative stress, and apoptosis in the relative sterility rat model following busulfan administration

**DOI:** 10.1186/s12906-022-03650-w

**Published:** 2022-06-23

**Authors:** Amirabbas Rostami, Sina Vakili, Farhad Koohpeyma, Bahia Namavar Jahromi, Ziba Afshari Aghajari, Fatemeh Mahmoudikohani, Forough Saki, Marzieh Mahmoodi, Khojaste Rahimi Jaberi, Ahmad Movahedpour, Majid Jafari Khorchani, Saam Noroozi

**Affiliations:** 1 Department of Internal Medicine, Faculty of General Medicine, Yerevan State Medical University after Mkhitar Heratsi, Yerevan, Armenia; 2 Infertility Research Center, Shiraz University of Medical Sciences, Shiraz, Iran; 3 Shiraz Endocrinology and Metabolism Research Center, Shiraz University of Medical Sciences, Shiraz, Iran; 4 Department of Obstetrics and Gynecology, School of Medicine, Shiraz University of Medical Sciences, Shiraz, Iran; 5 Radiation Oncology Department, Shiraz University of Medical Sciences, Shiraz, Iran; 6 Department of Midwifery, School of Nursing and Midwifery, Bam University of Medical Sciences, Bam, Iran; 7 School of Nutrition and Food Sciences, Shiraz University of Medical Sciences, Shiraz, Iran; 8 Shiraz nephro-urology research center, shiraz university of medical sciences, Shiraz, Iran; 9 Behbahan Faculty of Medical Sciences, Behbahan, Iran; 10 Biochemistry Department, Tarbiat Modares University, Tehran, Iran; 11 Department of biochemistry, Fasa university of medical sciences, P.O. Box: 7193635899, Shiraz, Iran

**Keywords:** Ellagic acid, Testicular tissue, Sterility, Rat, Sex hormones

## Abstract

**Background:**

Busulfan is an antineoplastic medication that is broadly utilized for cancer treatment. It affects the testicular function and leads to sterility. The present study aimed to evaluate the effects of ellagic acid on testicular tissue changes, sexual hormones, antioxidant defense system, and caspase-9 and Bcl2 gene expression in the busulfan-induced relative sterile rat model.

**Methods:**

This is an interventional-experimental animal study that was performed on 65 Adult male rats; they were randomly divided into five groups including control (1 ml of 0.9% normal saline), ellagic acid (50 mg/kg); busulfan (10 mg/kg); and busulfan plus ellagic acid (10 mg/kg and 50 mg/kg). At the end of the experiment, blood samples were collected, and plasma levels of sex hormones, antioxidant system, apoptosis-related genes, and testis histology were assessed.

**Results:**

Busulfan reduced the levels of serum testosterone, total antioxidant capacity, gene expression of Bcl2, testicular volume, seminiferous tubule, germinal epithelium, interstitial tissue volume, and the number of spermatogonia, spermatocyte, round spermatid, elongated spermatid, Sertoli cells and Leydig cells (*p <* 0.05). Busulfan administration resulted in a significant increase (*p <* 0.05) in the level of LH, FSH, malondialdehyde, and caspase 9. Busulfan + ellagic acid (50 mg/kg) showed higher serum levels of testosterone, gene expression of Bcl-2 and antioxidant markers, and lower LH, FSH levels, and gene expression of caspase 9 compared to the Busulfan-treated rats (*p <* 0.05). Stereological parameters were also ameliorated in the group treated with Busulfan+ 50 mg/kg ellagic acid (*p <* 0.05).

**Conclusion:**

In conclusion, the consumption of ellagic acid may have beneficial effects on the antioxidant defense system, sexual hormone abnormality, and testicular tissue damage induced by busulfan.

## Background

Chemotherapy and radiation are associated with many changes in the reproductive system, among which alkylating agents cause the most adverse effects on the gonads [1]. It has been shown that almost 72.4 million couples around the world suffer from infertility. Also, the reported infertility rates were 3.3% in Iran [2]. Today, especially in developed countries, successful treatment of malignancy and the life expectancy of these individuals have notably increased, and most of them, especially young people, tend to be fertile after disease recovery [3].

Busulfan is one of the drugs used in chemotherapy that has alkylating properties and leads to enhanced oxidative stress, apoptosis, and necrosis; it finally decreases the activity of the gonads and increases the endocrine abnormality [1, 4, 5]. Furthermore, it is commonly used to treat chronic leukemia and ovarian cancer [6, 7]. This drug is part of the methane sulfonic acid di esters group called 1,4-butanediol dimethanesulfonate [8]. According to previous studies, the fetus or neonate of rats that were born from pregnant mothers who had been exposed to busulfan during pregnancy had gonadal dysfunction and reduced the testicular germ cells and somatic cells [9, 10]. Bahmanpour et al. (2017) have revealed that testicular stereological parameters such as the weight and volume of the testes; tubules volume density; interstitial tissue; germinal epithelium; the number of spermatogonia, spermatocyte, round and elongated spermatid; and the Sertoli and Leydig cells significantly reduced by busulfan treatment and its related induced oxidative stress [11]. On the other hand, previous research has indicated various adverse effects of busulfan injection on the male reproductive system including decreased epididymis volume, Oligospermia, increased apoptotic sperm, and changes in the serum levels of testosterone, induced oxidative stress, and cytotoxicity [12–14]. Administration of busulfan, as a single dose, in high doses (40-55 mg/kg body weight) in adult mice induces azoospermia [15, 16].

Chemotherapeutic drugs cause damage to the mitochondrial membrane by producing free radicals [17]. Overproduction of reactive oxygen species (ROS) plays a potential role in mitochondrial membrane damage and stimulation of the release of cytochrome c which leads to the initiation of the intrinsic apoptosis pathway in the testicular tissue cells [18]. It has been shown that ROS reacts rapidly with membrane lipids, thereby resulting in lipid peroxidation (LPO) and cell loss [19]. Moreover, excessive production of ROS could lead to DNA damage, endothelial injury, apoptosis, and necrosis of the germinal cells. Therefore, ROS leads to severe damage to the reproductive tissues [18, 20, 21]. The release of cytochrome C is controlled by the BCL-2 family proteins located in the inner mitochondrial membrane [22]. The BCL-2 family proteins are composed of two groups anti-apoptotic and pro-apoptotic. The anti-apoptotic group consists of BCL-2 and BCL-XL which inhibits the release of cytochrome C into the cytosol, and the pro-apoptotic group includes Bid, Bax, and Bad, which release the cytochrome C from the mitochondrial cytosol [22, 23].

Caspases are a variety of cysteine-aspartate enzymes of the apoptotic pathway that play an important role in regulating apoptosis. Caspases are classified into two types of initiation and functional, including caspases 8, 9, and 10, and caspases 3, 6, and 7, respectively. The outer pathway contains caspases 8 and 10 and the inner pathway contains caspase 9, both of which are convergent pathways and utilize functional caspases that cascade activated and lead to cell destruction. Thus, it can serve as a suitable candidate gene for the study [24–28].

Several methods are used to prevent infertility after chemotherapy including freezing testicular and ovarian tissue, enhancing the resistance of sex stem cells, and helping to maintain the activity or repair of these cells, including maintaining cells. Among them, maintaining cells during chemotherapy has been considered more important than other methods [29–31]. Therefore, the administration of antioxidants seems to be essential for reducing oxidative stress and detoxification of the tissues [32, 33]. One of the substances more considered by researchers is ellagic acid. Ellagic Acid (2, 3, 7, 8-Tetrahydroxy-Chromeno (3, 4, 5-Cd) Chromeno-5, 10-Dione), with a molecular weight of 302 g/mol, is a polyphenolic compound. It is a natural and potential antioxidant found in most fruits, seeds, and vegetables including green tea and other natural sources including pomegranate, strawberry, blackberry, walnut, and mango [34, 35]. Like other polyphenols [36, 37], it has various features including anticancer [38] and antioxidant properties [39]; it has a direct protective effect against oxidative damage and can neutralize oxidative reactions [40]. Ellagic acid increases the activity of the antioxidant enzymes such as catalase, superoxide dismutase, and glutathione peroxidase, which are altered in diseases caused by free radicals [41]. It also inhibits the production of free radicals mediated by iron in vitro [42]. Previous studies have shown that ellagic acid reduces the side effects of arsenic, cisplatin, tobacco smoke, and monosodium glutamate-induced testicular structural alterations in male rats [43–46].

We hypothesize that Ellagic acid improves the testicular function and structure in a rat model of sterility induced by busulfan. Hence, the present study aimed to investigate the effect of ellagic acid on sexual hormones (testosterone, luteinizing hormone (LH), and follicle-stimulating hormone (FSH)), antioxidant system, stereological changes, and Gene Expression of Bcl-2 and Caspase-9 in the relative sterile rat model following administration of busulfan.

## Methods

### Experimental animals

At the beginning of the experiment, 65 male Sprague-Dawley rats (3 months old, weighing 200–250 g) were purchased from the Laboratory Animals Research Center (Shiraz University of Medical Sciences, Iran). The sample size was estimated according to previous studies [11]. The animals were acclimatized to the laboratory conditions for 2 weeks before the initiation of the experiments. They were fed with rodent chow (Behparvar Co., Tehran, Iran) and water during the study. Rats were kept in stainless steel cages in a temperature-controlled (22–25 °C) environment with 12 hr. light/dark cycles and 55% humidity [47]. All of the protocols of the study were under ARRIVE and NIH guidelines for reporting animal experiments and were approved by the Institutional Animal Ethics Committee of Fasa University of Medical Sciences (Fars, Iran) with ID of 97,024.REC1397.105.

### Induction of relative sterility

The relative sterility rat model was induced by intraperitoneal administration of a single dose of 10 mg/kg busulfan (Pierre Fabre, France) according to a study conducted by Bahmanpour et al. (2017) [11].

### Experimental design

The rats were divided randomly into five groups of 13 rats per group.

Group 1, the control group, received 0.9% saline solution as a vehicle (orally once per day for 48 days).

Group 2, the ellagic acid group (E.A 50), received 50 mg/kg b.w ellagic acid [19] orally once per day for 48 days.

Group 3, the busulfan group (BUS), received a single dose of the intraperitoneal injection of 10 mg/kg of busulfan [11].

Group 4, the treatment group (BUS+ E. A 10), received a single dose of busulfan (10 mg/kg a single i.p. injection) + 10 mg/kg b.w ellagic acid [45] orally once per day for 48 days.

**Fig. 1:**
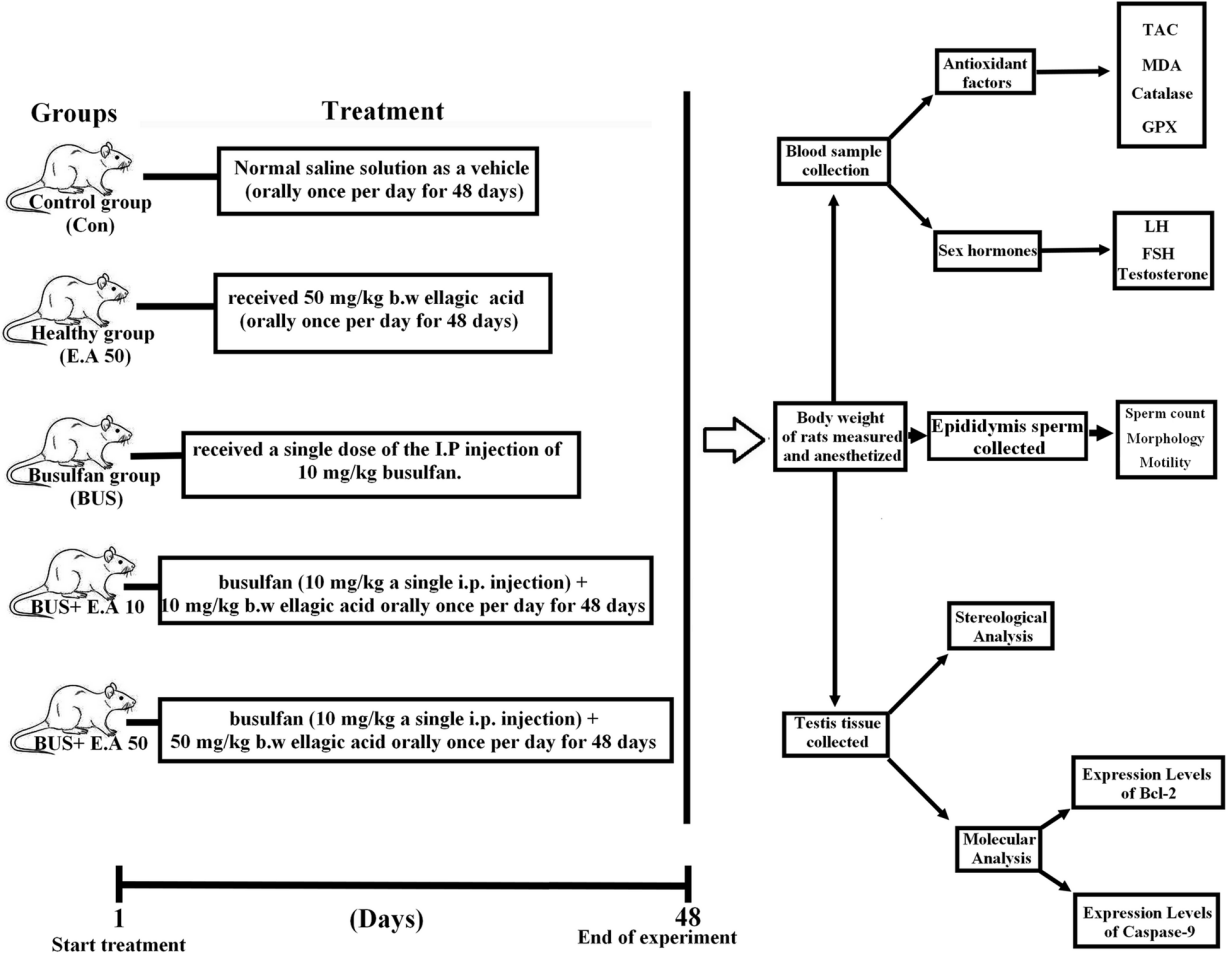
Schematic representation of the experimental design, showing the study groups and the timeline. I.P., intraperitoneal injection; E.A., Ellagic Acid; BUS: Busulfan

Group 5, the treatment group (BUS+ E. A 50), received a single dose of busulfan (10 mg/kg a single i.p. injection) + 50 mg/kg b.w ellagic acid orally once per day for 48 days (Fig. 1).

During the study, the animals were weighed by a digital scale (BAOSHISHAN ZQ-563, China) once a week. At the end of the 48-day treatment, after 12 hours of fasting and under anesthesia with ketamine (10%)/ xylazine (2%) mixture (80/5 mg/kg) (Alfasan, Netherland), 5 ml of blood was collected by cardiac puncture. In the present study, the animals were sacrificed by an overdose of sodium thiopental (100 mg/kg, intraperitoneal).

### Determination of biochemical parameters

The blood samples were centrifuged at 3500 rpm for 10 min to separate the sera and stored at − 80 °C before biochemical measurements. The sex hormones including testosterone, LH, and FSH were assessed by rat hormone ELISA kits (Bioassay Technology laboratory, BIOTECH company, China). Catalase activity and total antioxidant concentration (Zellbio Co, German) were measured using spectrophotometry [48] and glutathione peroxidase (GPX) enzyme activity by the Biorex kit (Fars, Iran). Serum malondialdehyde (MDA) concentrations were determined by a colorimetric method [49].

### RNA isolation and quantitative RT-PCR determination of Caspase-9 and Bcl-2 gene expression levels

The total RNA from the testicular tissue was isolated using the TRIzol reagent (Invitrogen), and the cDNA was synthesized following the manufacturer’s protocol, using 1 μg RNA (Prime Script™ RT reagent Kit, Takara). RT-PCR was done using a standard SYBR-Green PCR kit (SYBR Premix EX Taq™ II, Takara), and the gene-specific PCR amplification was conducted using the Applied Biosystems StepOnePlus™ Real-Time PCR System (Applied Biosystems, USA). The qRT-PCR reactions, including the no-template controls, were done in triplicate. Each PCR reaction was performed in a 20 μL solution containing 0.8 μL (10 μM) each of forward and reverse primers, 10 μL of Premix Ex Taq DNA polymerase, 0.4 μL of ROX reference dye, 6 μL of dH_2_O, and 2 μL of reverse transcription reaction products. The qRT-PCR primers used in the experiment are listed in Table 1. All experiments were performed in quadruplicate. Relative expression was determined by the 2^-ΔΔCt^ method using the housekeeping gene, GAPDH, as an internal control, and the fold change was calculated through comparison with the corresponding control group [50]. Primer sequences are demonstrated in Table 1.

**Table 1:** Gene specific-forward and reverse primer sequences

Primer	GC%	Length (bp)	TM	Sequences (5′- > 3′)	PCR Product length
*Cas9:F*	*55*	*20*	60.39	ACATCTTCAATGGGACCGGC	85 bp
*Cas9:R*	*52.38*	*21*	60.20	TCTTTCTGCTCACCACCACAG	
*GAPDH:F*	*50*	*20*	59.96	AAAGAGATGCTGAACGGGCA	100 bp
*GAPDH:R*	*47.62*	*21*	59.79	ACAAGGGAAACTTGTCCACGA	
*Bcl-2:F*	*50*	*20*	57.78	GGAGGATTGTGGCCTTCTTT	100 bp
*Bcl-2:R*	*50*	*20*	57.98	GTCATCCACAGAGCGATGTT	

### Stereological study

The left testicle tissue was separated from all the surrounding tissues and harvested; then, the weights of the testicles were calculated by scales, and the primary volume was determined using the immersion technique [51]. The Orientator method was used to acquire *Isotropic* uniform random. *In the next step, we put the slic*ed testes in paraffin molds, so that the trocar fragment was placed in the middle of the other parts. Then, we prepared 5-μm-thick sections for calculation of the volume density and 20-μm-thick sections for calculation of the number of cells. Tissue sections were dyed with Hematoxylin-Eosin (H&E) and Trichrome Masson [52]. After preparing the slides, stereology software was used for the analysis of the results.

The degree of shrinkage was assessed by the following formula based on the volume of the tissue [51, 52]:

$$\mathrm{Volume}\;\mathrm{Shrinkage}=1-\;{(\mathrm{Area}\;\mathrm{after}/\mathrm{Area}\;\mathrm{before})}^{1.5}$$

Then, the following formula was used to calculate the germinal epithelium, tubules, and interstitial space volume ratio [51, 52].

$$\mathrm{Vv}\left(\mathrm{structure}\right)=\sum_{\mathrm{i}=1}^{\mathrm{n}}\mathrm{p}\ \left(\mathrm{structure}\right)/\sum_{\mathrm{i}=1}^{\mathrm{n}}\left(\mathrm{reference}\right)$$

Where the “ΣP_Structure_” was the number of points hitting the profiles of the germinal epithelium or tubules or interstitial tissue, and “ΣP _references_” was the number of points hitting the testis. The method of calculation of numerical density and the absolute number of cells [51–53] was as follows:

$$\mathrm{Nv}=\frac{\sum_{\mathrm{i}=1}^{\mathrm{n}}\mathrm{Q}}{\sum_{\mathrm{i}=1}^{\mathrm{n}}\mathrm{P}\times \mathrm{h}\times \left(\frac{\mathrm{a}}{\mathrm{f}}\right)}\times \frac{\mathrm{t}}{\mathrm{BA}}$$

Where ΣQ was the number of the whole cells counted in all the dissectors, h was the height of the optical dissector, a/f was the area of the counting frame, Σp was the total number of the counted frames, BA was the microtome block advance to cut the block, and t was the mean of the final section thickness.

### Epididymis sperm analysis

The terminal part of the epididymis was removed from each rat, minced, and placed in a watch glass containing 5 ml of Ham’s F10 medium and kept in the incubator at 37 °C for 5 min. Afterward, one drop of solution was added to the Neubauer hemocytometer (Deep 0.1 mm, LAB ART, Germany). The number of sperms in four squares of the Neubauer chamber was counted. The mean was multiplied by 10^6^ to obtain the total number of sperm cells per mL of semen. For the assessment of sperm morphometry, after the preparation of the sperm smear, the slides were air-dried and then fixed with 96% ethanol. Then, the slides were stained with 1% eosin Y for 5-10 minutes and then left to dry. 100 spermatozoa were counted per rat for each sample, and the percentage of abnormal spermatozoa was specified. To calculate the sperm motility, the motility of 100 spermatozoa was assessed in ten microscopic fields at × 40 magnification, and then the mean of sperm motility was reported as a percentage. Spermatozoa motility was classified as 1: rapid progressive when spermatozoa moved rapidly and linearly; 2: immotile when spermatozoa showed no movement [54].

### Statistical analysis

Statistical analysis was done using SPSS software, version 23 (SPSS Inc., Chicago IL). Data were expressed as mean ± standard deviation (SD). the normally distributed data were compared between the groups by one-way ANOVA test (and Tukey test as post hoc), and non-normally distributed data were compared using the Kruskal-Wallis test (and Mann–Whitney U-test as post hoc). A *P*-value of < 0.05 was considered statistically significant.

## Results

### Effects of ellagic acid on body weight in busulfan-induced relative sterility in rats

Bodyweight significantly decreased (*P* = 0.001) in the BUS group compared to the control and E.A.50 groups. Our findings showed that there was a significant increase in the body weight in the BUS+E.A.50 group (*P* = 0.011) compared to the BUS group. Also, there were no significant changes in the body weight in E.A.50 treated group compared to the control group (Table 2).

**Table 2:** Evaluation of the body weight, LH, FSH, and testosterone concentrations in experimental groups

Group	Body weight (g)	LH (mIU/ml)	FSH (mIU/ml)	TES (nmol/L)
Con	337.75 ± 33.16 ^**a**^	21.68 ± 2.93^**a**^	24.34 ± 2.21^**a**^	96.32 ± 14.78^**a**^
E.A.50	337.37 ± 33.76 ^**a**^	20.19 ± 1.09^**a**^	23.60 ± 3.20^**a**^	96.60 ± 11.00^**a**^
BUS	256.87 ± 36.87 ^**b**^	36.98 ± 1.28^**b**^	40.58 ± 4.68^**b**^	42.73 ± 9.30^**b**^
BUS+ E.A.10	305.85 ± 50.45 ^**ab**^	29.04 ± 5.89^**c**^	33.08 ± 3.89^**b**^	60.69 ± 3.92^**bc**^
BUS+ E.A.50	323.25 ± 34.66 ^**a**^	23.80 ± 1.78^**ac**^	25.46 ± 3.79^**a**^	76.99 ± 11.16^**ac**^

^a^, ^b^, ^c^, ^ab^, ^ac^, and ^bc^: According to post-hoc Tukey test which was used for intergroup comparisons, groups with the same superscripts are not significantly different at α = 0.05 (*p* ≥ 0.05). However, dissimilar letters indicate a significant difference (*p* < 0.05)

### Effects of ellagic acid on sexual hormones in busulfan-induced relative sterility in rats

The concentration of LH and FSH significantly increased in the BUS group compared to the control group (*P <* 0.001), while that of testosterone decreased in the busulfan treated group when compared to the control group (*P <* 0.001). In addition, LH and FSH concentrations in EA. 50 and BUS+EA.50 groups were similar to the control, (*p <* 0.05). The testosterone levels in the EA50 and BUS+EA.50 groups were similar to those in the control rats (*p <* 0.05) (Table 2).

### Effects of ellagic acid on antioxidant parameters in busulfan-induced relative sterility in rats.

**Fig. 2:**
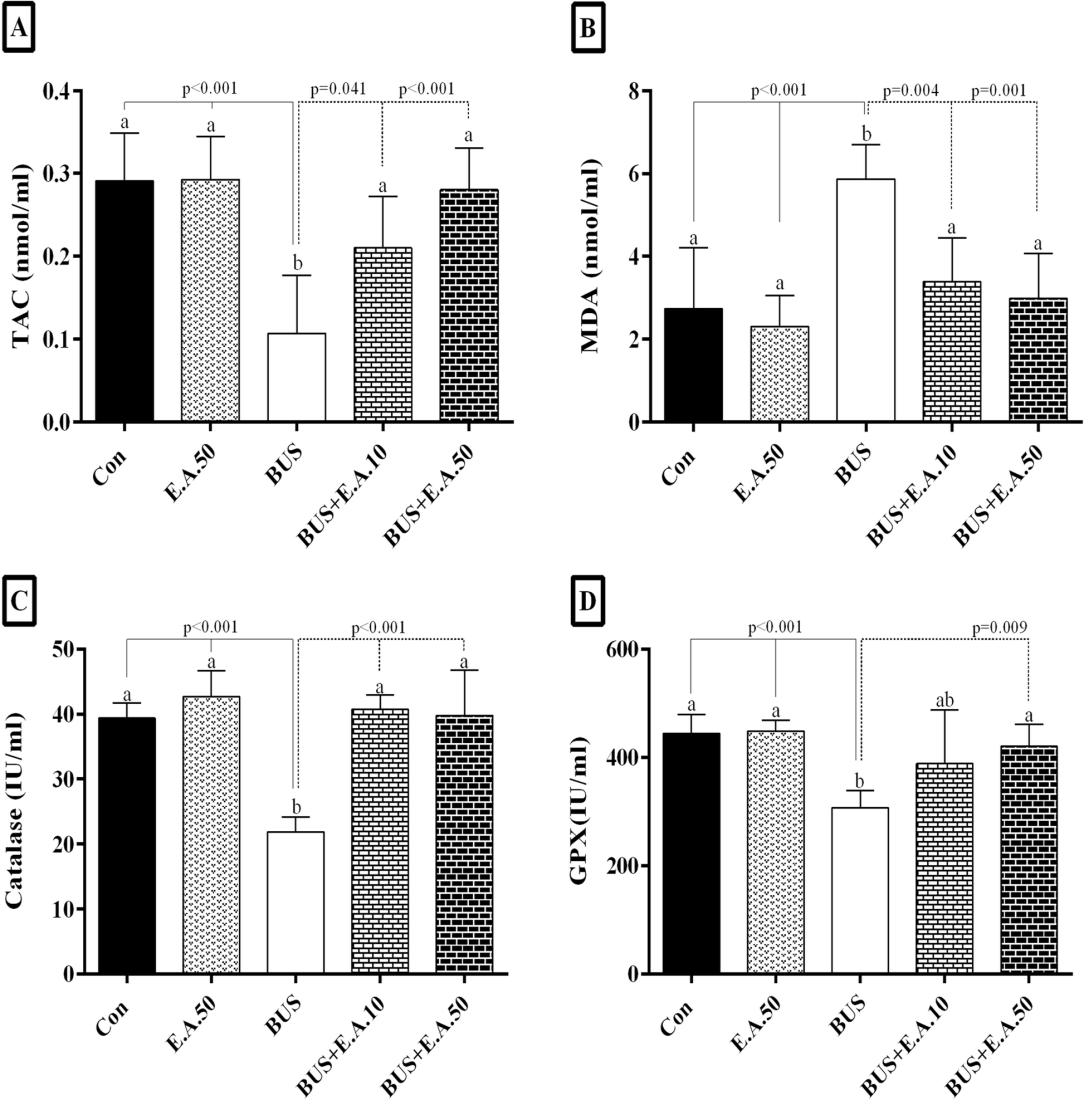
Comparison of TAC (**A**), MDA (**B**), catalase (**C**), and GPX (**D**) levels in the experimental groups. The results are presented as mean ± SD. According to the post-hoc Tukey test which was used for intergroup comparisons, groups with the same superscripts (a, b, and ab.) are not significantly different at α = 0.05 (*p* ≥ 0.05). However, dissimilar letters indicate a significant difference (*p <* 0.05)

A significant decrease in TAC, catalase, and GPX levels was observed in the BUS group compared to the control group (*P <* 0.001). Also, the MDA level in the BUS group was significantly higher than in the healthy group (*P <* 0.001). TAC and catalase activity was significantly increased in the BUS+ E.A.10 and BUS+ E.A.50 groups compared to the BUS group (*P <* 0.01). MDA concentration was significantly decreased in the BUS+ E.A.10 and BUS+ E.A.50 groups compared to the BUS group (*P <* 0.001). Also, the GPX activity was significantly increased in the BUS+E.A.50 compared to the BUS group (*P* = 0.009) (Fig. 2).

### Effects of ellagic acid on the mRNA expression levels of Bcl-2 and caspase- 9 in busulfan-induced relative sterility in rats

**Fig. 3:**
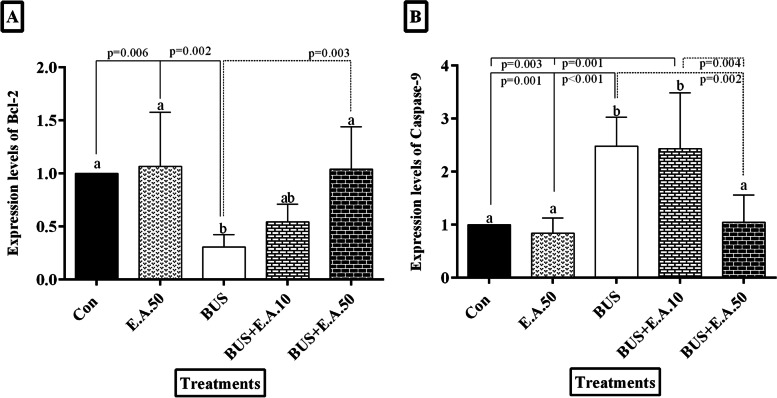
The effect of treatment with ellagic acid on mRNA expression. **A** Levels of Bcl-2, and (**B**) Caspase-9. Data are presented as mean ± SD. According to the post-hoc Tukey test which was used for intergroup comparisons, groups with the same superscripts (a, b, and ab.) are not significantly different at α = 0.05 (p ≥ 0.05). However, dissimilar letters indicate a significant difference (*p <* 0.05)

Gene expression of Bcl-2 was significantly decreased in the BUS group than in the control rats (*P* = 0.004). Bcl-2 gene expression was significantly increased in the BUS+ E.A.50 group compared to the BUS group (*P* = 0.006). However, Bcl-2 expression in the rats that received BUS + EA.50 was similar to that in the control rats (*p* > 0.05). Moreover, gene expression of Caspase-9 level was significantly increased in the BUS group than in the control and EA.50 groups (*P* = 0.002). Also, caspase-9 expression in the BUS+ E.A.50 group was similar to the control groups 1 and 2 (*p <* 0.05) (Fig. 3).

### Effects of ellagic acid on stereological parameters in busulfan-induced relative sterility in rats

#### The weight and volume of testis

**Fig. 4:**
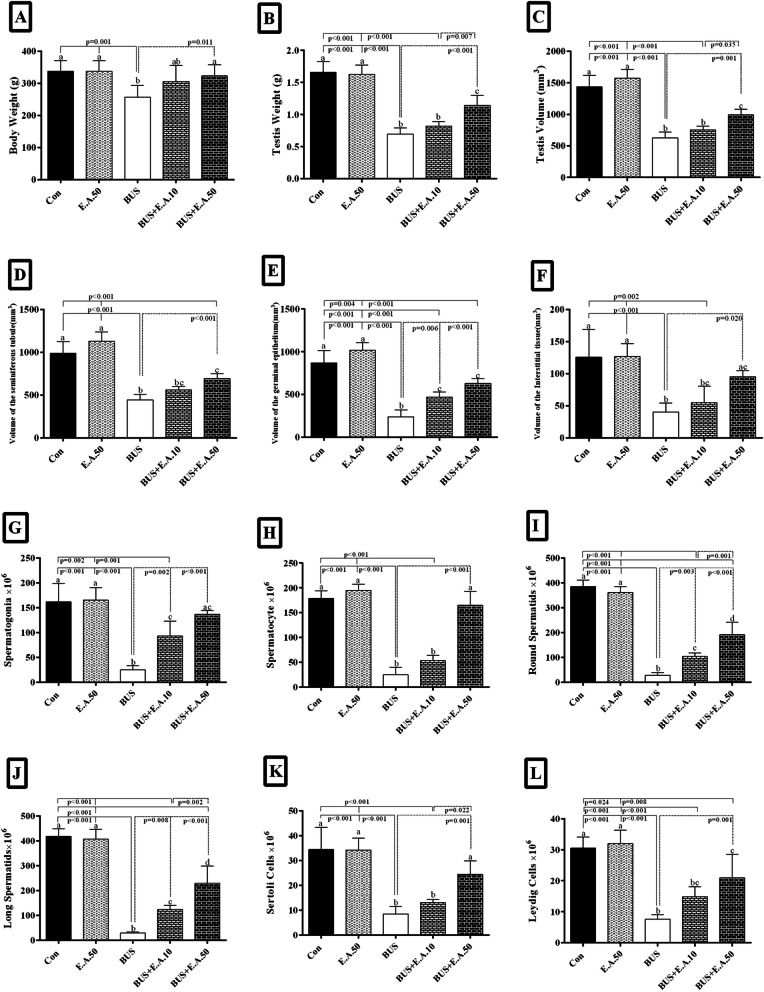
Evaluation of the body weight and the stereological parameters of the testis after 48 days of treatment. The column graph of the body weight (**A**), testis weight (**B**), the volumes of the testicle (**C**), seminiferous tubules (**D**), the Germinal epithelium (**E**) and interstitial tissue (**F**), and the number of spermatogonia (**G**), spermatocytes (**H**), round spermatids (**I**), elongated spermatids (**J**), Sertoli (**K**) and Leydig (**L**) in the experimental groups. Data are presented as mean ± SD. a, b, c, d, ab, ac, and bc. According to the post-hoc Tukey test used for intergroup comparisons, groups with the same superscripts are not significantly different at α = 0.05 (p ≥ 0.05). However, dissimilar letters indicate a significant difference (*p <* 0.05)

Testicular weight and volume in the BUS group were decreased by about 58.2 and 56.4%, respectively, compared to the control group (*p* < 0.001). Testicular weight and volume in BUS+E.A50 was 39.94 and 36.83%, respectively, higher than the BUS (*p* ≤ 0.001). No significant difference in the testicular weight and volume between the BUS and BUS+E.A.10 groups. The results indicated that testicular weight and volume in the BUS+E.A.50 group were 28.59% and 23.91, respectively, higher than in the BUS+E.A.10 group (*p* = 0.007, *p* = 0.035). However, testicular weight and volume in the BUS rats treated with *E.A.50* were lower than those in the control group (*p <* 0.001) (Fig. 4).

#### Seminiferous tubule, germinal epithelium, and interstitial tissue volume

**Fig. 5:**
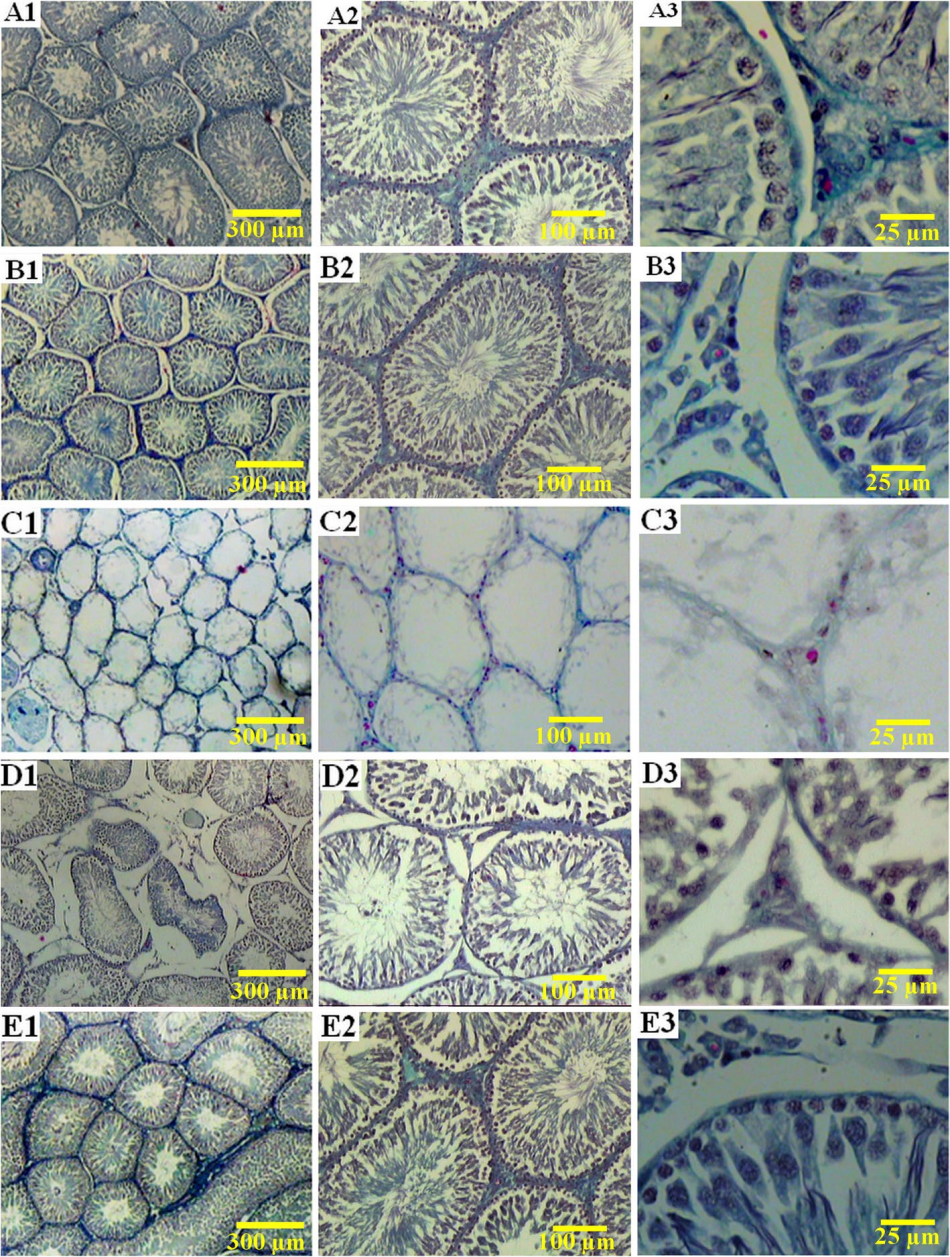
Photomicrograph of the histology of the testicles in different groups. **A1**, **A2**, **A3** the control rats with normal structure seminiferous tubules, interstitial tissue, and the number of sexual linage cells. **B1**, **B2**, **B3** the healthy group (E.A 50), which received 50 mg/kg ellagic acid with normal testis histopathological features. **C1**, **C2**, **C3** the busulfan group: the seminiferous tubules appeared atrophic, the germinal epithelium height was destroyed, and many testicular cells were lost. **D1**, **D2**, **D3** azoospermia rats treated with ellagic acid 10 mg/kg showed fewer pathological changes and improved testis architecture. **E1**, **E2**, **E3** the sexual cell population significantly ameliorated in the rats treated with ellagic acid 50 mg/kg compared to those that received busulfan. **A**-**E** Trichrome Masson staining with magnification at × 40, × 100, × 400

The results showed that in comparison to the control group, the volume of the seminiferous tubule, germinal epithelium, and interstitial tissue was reduced by 55.05, 72.41, and 67.80% in the BUS group, respectively (*p <* 0.001). Also, these parameters in the BUS+E.A.50 group were higher about 56.22%, 1.6 fold, and 1.3 fold, respectively, compared with the BUS group (*p <* 0.001, *p <* 0.001, *p* = 0.020). There is a significant increase in the germinal epithelium volume of the rats in the BUS+E.A.10 group compared with the BUS group (*p* = 0.006). In addition, the volume of the seminiferous tubule and the germinal epithelium volume in the BUS + E.A.50 rats were not within the range of the control group, showing a significant reduction (*p <* 0.001, *p* = 0.004) (Figs. 4, 5).

#### The sexual lineage cell number

There was a significant decrease in the number of spermatogonia (84.64%), spermatocyte (85.95%), round spermatid (92.69%), elongated spermatid (92.93%), Sertoli cells (75.18%), and Leydig cells (75.14%) in the BUS group compared to the control group. Busulfan-treated rats which received ellagic acid 10 and 50 mg/kg showed a significant increase in the number of spermatogonia (3, 4.5 fold), round spermatid (3, 5.7 fold), and elongated spermatid cells (3, 6 fold) (*p <* 0.01), compared to the BUS-treated rats. In addition, in the BUS rats, the number of spermatocytes, Sertoli, and Leydig cells treated with E.A.50 was significantly higher compared to the BUS groups (5.5 fold, 1.8 fold, and1.7 fold, respectively, *p* ≤ 0.001) (Fig. 4).

During the study, we did not find any side effects of ellagic acid in the rats. Also, there was no significant change in the body weight, LH, FSH, testosterone, TAC, MDA, catalase, GPX, Bcl-2, and Caspase-9 gene expression, testicular weight and volume, seminiferous tubule volume, germinal epithelium volume, interstitial tissue volume, and the number of spermatogonia, spermatocyte, round spermatid, elongated spermatid, Sertoli cells and Leydig cells in E. A50, as compared to the control group.

In summary, testis sections of the control and E.A.50 groups did not show any histopathological changes, and the testis architecture was normal. Some irregularity and shrinkage in the seminiferous tubules outline were revealed in the testicles of the busulfan-exposed rats. There was some degree of degeneration and apoptosis in the sexual lineage cells in the BUS group. In this group, the basement membrane and germinal epithelium were thickened and irregular, respectively; interstitial cells of Leydig had scanty cytoplasm with deeply stained vesicular nuclei. The testis of the busulfan rats treated with 10 mg/kg of ellagic acid showed little pathologic changes compared to the BUS group. However, fewer pathological changes and disruptions in the testicular architecture were observed in groups treated with ellagic acid 50 mg/kg (Fig. 5).

### Effects of ellagic acid on the sperm count, morphology, and motility in busulfan-induced relative sterility in rats

There was a significant decrease in the number of sperms (89.95%) and sperm progressive motility in the BUS group compared to the control group (*p <* 0.001), (Table 3). Also, there was no significant difference in the sperm count and sperm motility between the busulfan and the ellagic acid 10 groups (*p <* 0.001). Busulfan-treated rats which had received ellagic acid 50 mg/kg indicated a significant increase in the number of sperm (~ 3.7 fold) and sperm progressive motility (*p <* 0.001), compared to the BUS-treated rats. (Table 3). A significant increase in the percentage of abnormal morphology sperm was observed in the BUS group compared to the control group (*P <* 0.001). On the other hand, the percentage of abnormal morphology sperm was significantly decreased in the BUS+ E.A.10 and BUS+ E.A.50 groups compared to the BUS group (*P <* 0.001). There was a significant difference in the percentage of abnormal sperm between the BUS+ E.A.10 and the BUS+ E.A.50 recipient groups (Table 3).

**Table 3:** Sperm count, morphology, and motility of the sperm in the experimental groups

Group	Sperm count (×10^**6**^)	Abnormal morphology	% Sperm motility	
			Progressive motility	Immotile
**Con**	13.04 ± 1.08 ^**a**^	4.40 ± 2.30^**a**^	81.40 ± 4.93^**a**^	18.60 ± 4.93^**a**^
**E.A.50**	14.10 ± 1.52 ^**a**^	3.24 ± 1.62^**a**^	85.20 ± 2.86^**a**^	14.80 ± 2.86^**a**^
**BUS**	1.31 ± 0.43 ^**b**^	75.80 ± 8.89^**b**^	1.20 ± 0.45^**b**^	98.80 ± 0.45^**b**^
**BUS+ E.A.10**	2.14 ± 0.71 ^**b**^	57.40 ± 5.77^**d**^	4.80 ± 1.64^**b**^	95.20 ± 1.64^**b**^
**BUS+ E.A.50**	6.22 ± 1.63 ^**c**^	39.60 ± 4.09^**c**^	44.80 ± 3.56^**c**^	55.20 ± 3.56^**c**^

^a^, ^b^, ^c^, and ^d^: According to the post-hoc Tukey test which was used for intergroup comparisons, groups with the same superscripts are not significantly different at α = 0.05 (*p* ≥ 0.05). However, dissimilar letters indicate a significant difference (*p* < 0.05)

## Discussion

The results of the present study showed that busulfan significantly reduced testosterone, decreased the antioxidant parameters, reduced the testicular function (e.g. decreased germinal epithelium volume, seminiferous tubules volume, interstitial tissue volume, sexual lineage cells), and induced testicular cell apoptosis (e.g. reduced gene expression of Bcl-2 and significantly increased the plasma MDA level and gene expression of caspase-9). Therefore, these findings demonstrate the possibility of the relative sterility induction and death of germline cells after busulfan administration. It has been reported that busulfan contains 2 functional groups of sulfonated methane which can alkylate DNA on N7 and O6 of guanine, and N3 of adenine, forming intra-strand crosslinks at 50 -GA-30 and, to a lesser extent, at 50-GG-30. Therefore, DNA cross-linking, DNA–protein crosslinking, and single-strand breaks (SSBs) lead to the blockage of DNA replication and transcription, and inhibition of cell proliferation and differentiation [55].

Higher expression of p53 in response to DNA damage caused by busulfan [56] upregulates the adaptor protein, ASK (activator of S-phase kinase), which activates the expression of apoptosis target genes, such as Bax, Bid, and PUMA (p53 upregulated the modulator of apoptosis) [57]. These genes enhanced permeability and release of cytochrome C, thereby inducing apoptosis [58].

Moreover, Fas, induced by busulfan in the spermatogenic cells combines with procaspase-8 to form the death-inducing signaling complex (DISC) which activates caspase-8 [55]. Caspase 3 and caspase-7 lead to DNA cleavage and cell apoptosis by subsequently activated cleaving of the DNA repair enzyme, PARP (poly (ADP-ribose) polymerase), and increasing Ca^2+^/Mg^2+^-dependent endonuclease activity [55, 59], which is consistent with the findings of Hakemi et al. (2019), Dehghani et al. (2013) and Olfati’s et al.’s (2020) studies [60–62].

These findings confirmed the previous reports that showed toxic effects of busulfan in the rat testis, including changes in the count, morphology, motility of the sperm, and spermatogenesis [60, 61, 63]. In the present study, sperm motility and sperm count showed a significant decrease. On the other hand, sperm abnormality was higher in the busulfan-exposed rats. The ROS produced by busulfan can attack and damage bio-molecules such as DNA and lipids. The plasma membrane of the sperm has a great content of polyunsaturated fatty acids, so sperms are highly susceptible to oxidative stress. The effects of ROS produced by busulfan are disturbing the sperm membrane tail fluidity and reducing the sperm motility because of polyunsaturated fatty acids in the tail membrane of the sperm cell [64] which is similar to the present study results. Also, previous studies showed that the length of the sperm flagella was shortened in the rats that received busulfan, leading to decreased sperm motility [60, 61].

However, our results indicate that administration of 50 mg/kg of ellagic acid could improve the secretion of the sexual hormones, antioxidant and stereological parameters, and apoptotic gene expression changes in the rats with relative sterility. Moreover, similar to the study by Hosseini Ahar et al., we showed that the use of busulfan could reduce the body weight and testicular weight in male rats [65]. A study found that Ellagic acid ameliorated the body and testis weights of the animals. It has been reported that Ellagic acid at a 50 mg/kg dose has a protective effect [66], which is similar to the present study results. Moreover, it is in agreement with the report showing that cisplatin-induced decrease in the deteriorated histopathologic findings of the testis was partially ameliorated by ellagic acid treatment [46].

In the present study, ellagic acid also reversed the hypergondotropic-hypogonadism induced in the busulfan-treated rats, demonstrated by decreasing FSH and LH levels. It seems that improving the testicular function and structure induced by Ellagic acid could increase the testosterone secretion and *seminiferous* tubules volume resulting in lowering the LH and FSH secretion, respectively [67]. It has been shown that busulfan causes changes in the gonadotropin levels due to its destructive effects on the testicular tissue. In other words, it refers to the feedback effect on gonadotropin secretion in response to the gonadal damage [68, 69] that leads to increased FSH and LH levels [67].

Our major findings showed that ellagic acid potentially augmented the antioxidant enzymes such as catalase and ameliorated the MDA level. Ellagic acid is a natural phenol compound with a polyphenolic structure that has a DPPH-free radical scavenging activity and inhibits lipid peroxide production [70]. The cryoprotective and antioxidative properties of ellagic acid have been previously reported in the reduction of the lipid peroxidation and increment of the total glutathione (tGSH) levels in rats [71]. Other studies also reported the anti-oxidative properties of ellagic acid against oxidative stress [72].

Chemotherapy drugs can induce apoptosis in the germ cells of the testicular tissue [73]. In this study, the administration of ellagic acid ameliorated the apoptotic condition induced by busulfan. It is in agreement with the report that cisplatin-induced decrease in the germinal cell layer thickness and the deteriorated histopathologic findings of testis were partially ameliorated by ellagic acid treatment [46]. Ellagic acid has potential anti-apoptosis and anti-inflammatory effects [74]. These results are in the same line as those of Çeribasi et al., who reported the effects of ellagic acid on adriamycin-induced high lipid peroxidation levels and apoptosis in rats [71]. Bcl-2 is a key factor in the inhibition of apoptosis and its over-expression can effectively prevent the apoptosis induced by hydrogen peroxide, free radicals, and microbial contamination [74]. In this view, several studies have shown that oxidative DNA damage induced by the free radical attack can induce apoptosis [75, 76]. Yu et al. also demonstrated that ellagic acid suppressed apoptosis through its antioxidant effects [77]. In line with these findings, our results demonstrated that ellagic acid improved the abnormal gene expression level of Bcl-2 and caspase-9.

In the present study, the effect of ellagic acid administration on spermatogenesis was investigated. Administration of ellagic acid for 48 days reduced the adverse effects of busulfan on spermatogenesis. Therefore, it seems that the improvement in spermatogenesis is due to the antioxidant activity of Ellagic acid. The study carried out by Motlag et al. showed that ellagic acid could prevent the reduction of spermatogonia, Leydig and Sertoli cells as well as the diameter of spermatozoa tubules in the testicular tissue of the rats exposed to cadmium chloride [78], which is similar to our results. Additionally, in the present study, treatment with ellagic acid significantly increased spermatogenic lineage which leads to a significant increase in the thickness of the seminiferous tubule epithelium and diameter. Utomo et al. also reported that anthocyanin had a key role in ameliorating the oxidative stress that leads to maintaining the normal spermatogenesis and following preserve the spermatogenic cells [79]. Hence, enhanced epithelium thickness is related to increased numbers of Sertoli and spermatogenic cells [79], which is similar to our results.

Previous studies showed that Ellagic acid at doses of 10, 25, and 50 mg/kg was found to be effective in preventing sperm abnormalities in a dose-dependent manner [66]. Even though only partial protection was noted at lower doses, a 50 mg/kg dose of ellagic acid produced complete normalization of the studied parameters. The mechanism involved in the protective effect of ellagic acid against sodium busulfan-induced reproductive toxicity is unknown. Busulfan can disrupt the cellular mechanisms in several ways which can cause toxicity. It is going to induce radical formation and lipid peroxidation, which are chemical mechanisms capable of disrupting the structure and performance of the testis. The antioxidant and radical scavenging properties of ellagic acid may play a crucial role in preventing the toxic effects of the medicine [66]. Busulfan has been shown to possess an apoptosis-promoting effect on the rat testis by increasing caspase3 activity [62]. The compounds that have anti-apoptotic properties like ellagic acid could also be beneficial against gonadotoxins [80]. Future studies are required to explore the precise protective mechanism of ellagic acid. It is suggested that special observation tests, covering deoxynucleotidyl transferase dUTP Nick-End-Labeling (TUNEL) and BAX/Bcl-2 immunoblotting are required to confirm the occurrence of apoptosis in the testis tissue. That could be a related limitation that can be considered in future studies. It seems that ellagic acid inserts its positive role in preserving spermatogenesis through its antioxidant characteristics [81]. So clinical trials should be performed to clarify the exact mechanism of ellagic acid in humans and assess its possible improving effects as a complementary medicine for preventing infertility in busulfan-treated patients.

## Conclusion

The results demonstrated that the consumption of ellagic acid may have beneficial effects on the antioxidant defense system, sexual hormones abnormality, and testicular tissue function in busulfan-induced sterility in rats.

## Data Availability

The datasets used during the current study are available from the corresponding author on reasonable request.
